# Serum uric acid and risk of diabetic neuropathy: a genetic correlation and mendelian randomization study

**DOI:** 10.3389/fendo.2023.1277984

**Published:** 2023-11-15

**Authors:** Youqian Zhang, Zitian Tang, Ling Tong, Yang Wang, Lin Li

**Affiliations:** ^1^ Department of Endocrinology, The First Affiliated Hospital of Yangtze University, Jingzhou, Hubei, China; ^2^ Law School, Yangtze University, Jingzhou, Hubei, China; ^3^ Department of Neurology, Second Affiliated Hospital of Zhengzhou University, Zhengzhou, China

**Keywords:** diabetic neuropathy, serum urate acid, Mendelian randomization study, linkage disequilibrium score regression (LDSC), genetic correlation

## Abstract

**Background:**

Previous observational studies have indicated an association between serum uric acid (SUA) and diabetic neuropathy (DN), but confounding factors and reverse causality have left the causality of this relationship uncertain.

**Methods:**

Univariate Mendelian randomization (MR), multivariate MR and linkage disequilibrium score (LDSC) regression analysis were utilized to assess the causal link between SUA and DN. Summary-level data for SUA were drawn from the CKDGen consortium, comprising 288,648 individuals, while DN data were obtained from the FinnGen consortium, with 2,843 cases and 271,817 controls. Causal effects were estimated primarily using inverse variance weighted (IVW) analysis, supplemented by four validation methods, with additional sensitivity analyses to evaluate pleiotropy, heterogeneity, and result robustness.

**Results:**

The LDSC analysis revealed a significant genetic correlation between SUA and DN (genetic correlation = 0.293, P = 2.60 × 10^-5^). The primary methodology IVW indicated that each increase of 1 mg/dL in SUA would increase DN risk by 17% (OR = 1.17, 95% CI 1.02-1.34, P = 0.02), while no causal relationship was found in reverse analysis (OR = 1.00, 95% CI 0.98~1.01, *P* = 0.97). Multivariate MR further identified that the partial effect of SUA on DN may be mediated by physical activity, low density lipoprotein cholesterol (LDL-C), insulin resistance (IR), and alcohol use.

**Conclusion:**

The study establishes a causal link between elevated SUA levels and an increased risk of DN, with no evidence for a reverse association. This underscores the need for a comprehensive strategy in DN management, integrating urate-lowering interventions with modulations of the aforementioned mediators.

## Introduction

Diabetes mellitus (DM), a prominent metabolic disorder characterized by chronic hyperglycemia (1, 2), is increasingly widespread, with the World Health Organization (WHO) estimating over 422 million global cases. By the year 2045, this figure is projected to rise to 629 million (3, 4). As the paramount complication arising from DM, Diabetic Neuropathy (DN) demonstrates an elevated prevalence, exceeding 50%, in the populace diagnosed with this metabolic condition (5, 6). Diabetic polyneuropathy (DPN) and Peripheral Diabetic Neuropathy (PDN) are the most prevalent manifestations of DN (7). People living with DN experience a significant economic loss in addition to the medical costs of the disease due to missed opportunities at work and lost pay. According to a U.S. study on the financial consequences of diabetic neuropathy, the yearly immediate expense for each people is $4,841, and the quarterly supplementary expense is $9,730 (8). According to a study, the annual cost of healthcare for persons with painful DN is about three times higher than the cost for comparable control populations (9). Despite the clinical and financial costs associated with DN, there is no cure. Thus, the exploration of additional modifiable risk factors for DN is critical to enhance clinical management and prevent DN onset and progression.

Serum urate acid (SUA), which is the terminal product of purine metabolism, is under the regulatory influence of the enzyme xanthine oxidase. High SUA levels have been associated with vascular dysfunction and irreversible damage, potentially leading to tissue ischemia and compromised peripheral nerve function (10). Numerous investigations propose an associative relationship between augmented serum urate concentrations and the escalated incidence of DN (11–14). However, the evidence supporting this association is debatable. Contrasting investigations have found inconsistent associations between levels of SUA and DN. One study using data from the National Health and Nutrition Examination Survey (NHANES) discerned no consequential correlation between moderately increased urate levels and the peril of PDN, after adjustments for multivariate factors (15). Meanwhile, another study engaging 1,784 male and 1,025 female participants established that escalated SUA concentration constituted an autonomous risk determinant for non-alcoholic fatty liver disease and diabetic nephropathy. Nevertheless, no association was discernible with DPN (16).

Antecedent scholarly exploration failed to unequivocally substantiate the causal liaison between exposure constituents and outcome variables, due to the intricate scenarios engendered by confounding elements and the phenomenon of reverse causality. In light of these inconsistencies and the limitations of observational studies in determining causality, genetic research methods such as Mendelian randomization (MR) can offer valuable insights. Experiments utilizing MR apply genetic variations, identified via genome-wide association analyses, as instrumental variables (IVs). These IVs aid in estimating the cause-and-effect relationship between environmental exposure and the intended outcome. This methodology allows for causality inference under certain circumstances, using the genetic variants as stand-ins for environmental exposure (17). Conceptualized as a natural randomized controlled trial, MR operates on Mendelian laws of inheritance, assigning parental alleles to progeny. This method delivers a higher degree of evidence and a lower susceptibility to confounding factors. Compared to observational epidemiological research, MR has a greater level of evidence. Therefore, conducting a bidirectional MR study could play a pivotal role in uncovering the elusive causal relationships between SUA and DN, potentially leading to more effective preventative measures and treatments.

## Materials and methods

### Study design

**Figure 1:**
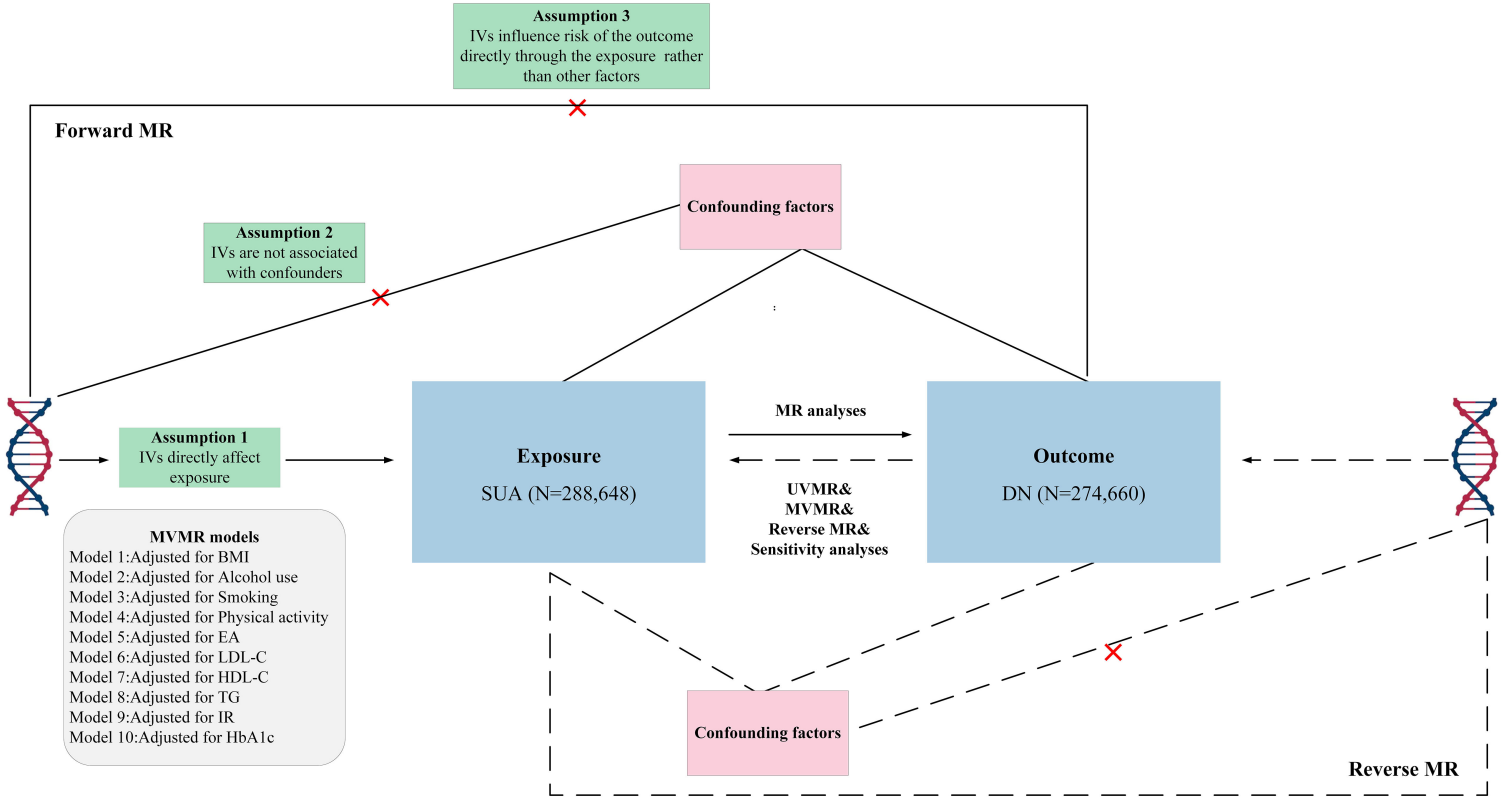
Overview of research design and analysis strategy. Overview of the research design. Exposures come from SUA, with outcomes including DN. The MR framework is based on three fundamental MR assumptions, with MVMR analyses adjusting for ten mediating factors for positive results. MVMR, multivariate Mendelian randomization; UVMR, Univariate Mendelian randomization; BMI, body mass index; EA, education attainment; DN, diabetic neuropathy; SUA, Serum urate acid; HbA1c, Glycated Hemoglobin A1c; LDL-C, Low Density Lipoprotein Cholesterol; HDL-C, High Density Lipoprotein Cholesterol; TG, Triglyceride; IR, insulin resistance.

To investigate the potential causal relationships between SUA concentrations and the risk of DN, bidirectional univariate MR (UVMR) and multivariable MR (MVMR) analyses were developed. (**Figure 1**). This study posited SUA as the exposure, DN as the outcome variable. The choice of IVs for serum urate levels hinged on three crucial assumptions: (i) the selected genetic variant, acting as the instrumental variable, demonstrates a robust association with the exposure; (ii) the genetic variant maintains no connection with potential confounders; and (iii) the influence of genetic variants on the outcome is mediated exclusively via the exposure, eliminating the possibility of alternate pathways (18). Conversely, we probed the reciprocal impact of DN on SUA, acknowledging the potential for reverse causation. Consequently, to ensure a robust correlation between all instrumental variables and the exposure, only SNPs demonstrating a genome-wide significance with SUA levels were considered. Furthermore, these genetic variants needed to display independence and avoid linkage disequilibrium, signifying their random allocation at conception. Given the potential for horizontal pleiotropy, additional analyses leveraging alternative statistical methodologies were conducted. In addition, as body mass index (BMI), alcohol use, smoking, education attainment (EA), and physical activity may play confounding roles in the exposure to outcome pathway, further bidirectional MVMR analyses were conducted to estimate the direct causal effect of exposure on outcome. Compared with the UVMR hypothesis, assumption 1 of the MVMR refers to genetic variation linked with one or more exposures, and the remaining assumptions are aligned with the UVMR (19).

### Data sources

DN (2,843 ncase and 271,817 ncontrol) data were obtained from the FinnGen consortium. The DN were defined by ICD-10 codes, with the phenotype adjusted for age, sex, and up to 20 genetic principal components. Instituted in Finland in 2017, the FinnGen study embarked on an expedition to collate and scrutinize genomic and health-related data from an expansive cohort of approximately half a million Finnish individuals (https://www.finngen.fi/en).

The data for SUA was sourced from the CKDGen consortium. The aggregated statistical data used in this study encompassed a total of 74 cross-ethnic studies involving 457,690 individuals, of which 288,649 were of European ancestry (20). The study adjusted for key components such as age, gender, and ancestry in the Genome-wide association studies (GWAS) meta-analysis and identified 183 urate-related genetic loci, with 147 being novel discoveries. Concurrently, the research designated a genetic urate risk score and significantly enhanced the prediction of gout risk for 334,880 participants.

Furthermore, we obtained genetic associations for BMI from the Genetic Investigation of Anthropometric Traits (GIANT) consortium (21), low density lipoprotein cholesterol (LDL-C), high density lipoprotein cholesterol (HDL-C), triglyceride (TG) from the Global Lipids Genetics Consortium (GLGC) (22), glycated hemoglobin A1c (HbA1c) from the Meta-Analyses of Glucose and Insulin-related traits Consortium (MAGIC) (23), insulin resistance (IR) from the Dupuis J et al (24), alcohol use from the Psychiatric Genomics Consortium (PGC) (25), EA from the GWAS of 1.1 million individuals conducted by the Social Science Genetic Association Consortium (SSGAC) (26), physical activity from the family GWAS consortium (27) and smoking were identified from the GWAS and Sequencing Consortium of Alcohol and Nicotine use (GSCAN), involving 1.2 million individuals (28).

A detailed presentation of the summary statistics from the data sources is available in **Table 1**; **Supplementary File 1**. Each investigation incorporated within the GWAS framework was sanctioned by the relevant ethical review panels. Written informed consent was procured from every participant involved. The data utilized in the present study maintain public accessibility.

**Table 1:** Detailed information of data sources.

Explore or Outcome	Ref	Ieu id	Consortium	Ancestry	Participants
Phenotypes					
Diabetic Neuropathy	NA	NA	FinnGen	European	2,843 cases / 271,817 controls
Serum urate acid	31578528	NA	CKDGen	European	288,649 individuals
Adjustment of the model					
LDL-C	24097068	ieu-a-300	GLGC	96% European	173,082 individuals
HDL-C	24097068	ieu-a-299	GLGC	96% European	187,167 individuals
TG	24097068	ieu-a-302	GLGC	96% European	177,861 individuals
HbA1c	20858683	ieu-b-104	MAGIC	European	46,368 individuals
IR	20081858	ebi-a-GCST005179	Dupuis J et al	European	37,037 individuals
Smoking	30643251	ieu-b-4877	GSCAN	European	311,629 cases / 321,173 controls
Alcohol use	30336701	NA	PGC	European	141,932 individuals
Physical activity	35534559	ieu-b-4860	The Within Family	European	78,007 individuals
BMI	30239722	NA	GIANT	European	694,649 individuals
EA	30038396	ieu-a-1239	SSGAC	European	1,131,881 individuals

BMI, body mass index; GSCAN, GWAS and Sequencing Consortium of Alcohol and Nicotine use; GIANT, Genetic Investigation of Anthropometric Traits; GLGC, Global Lipids Genetics Consortium; MAGIC, Meta-Analyses of Glucose and Insulin-related traits Consortium; LDL-C, Low Density Lipoprotein Cholesterol; HDL-C, High Density Lipoprotein Cholesterol; TG, Triglyceride; IR, insulin resistance; PGC, Psychiatric Genomics Consortium; EA, Education Attainment; SSGAC, Social Science Genetic Association Consortium; HbA1c, Glycated Hemoglobin A1c; Ref, reference(Pubmed id). NA, Not Applicable.

### Genetic instrument selection

Within the framework of our MR studies, we treated the included SNPs as genetic IVs. To ensure the accuracy of the MR estimates, these SNPs were required to satisfy specific criteria:

(1) All SNPs chosen as IVs manifested a correlation with the respective exposure at a genome-wide significance threshold (*P*<5×10^-8^). (2) SNPs were ensured to be unassociated with any potential confounders and independent of one another to prevent biases stemming from linkage disequilibrium (r^2^ < 0.001, clumping distance = 10,000 kb); (3) F-statistics were employed to test for weak instrumental variables, and all of the F-statistics of the incorporated SNPs exceeded 10. A larger F-statistic indicates stronger instrument strength. We calculated an F-statistic (F=beta^2^/se^2^; beta for the SNP-exposure association (beta); variance (se)) for each SNP (29). (4) To maintain the robustness of the results, proxy SNPs were not used and MR-Steiger filtering was employed to eliminate variations demonstrating stronger correlations with outcomes than with exposures (30).(5) The effect of an SNP on exposure and the effect of that SNP on outcome must each correspond to the same allele. (6) If an SNP is absent in the outcome dataset, we employ the SNiPa online tool (http://snipa.helmholtz-muenchen.de/snipa3/) to locate the respective SNP. We calculated the variance explained by each assay SNP. This tool utilizes European population genotype data derived from Phase 3 of the 1000 Genomes Project. Subsequently, another SNP exhibiting linkage disequilibrium (r^2^ > 0.8) with the initial SNP is identified as a proxy SNP.

### LDSC regression analysis

The linkage disequilibrium score (LDSC) regression analyses was applied to summary-level GWAS data to ascertain genetic correlations between the two distinct phenotypes. Initial stages of analysis involved employing munge_sumstats.py (https://github.com/bulik/ldsc/blob/master/munge_sumstats.py) to restructure summary statistics, and to eliminate variants that do not align with SNPs, such as indels, ambiguous strand SNPs, and duplicated SNPs. As a subsequent step, aligning with the methodology proposed by the original developers, we used the 1000 Genomes Project as the linkage disequilibrium (LD) reference panel to compute the LD score. In the final stage, the LDSC tool (https://github.com/bulik/ldsc) facilitated the assessment of genetic correlation between SUA, DN, and DPN.

### Statistical analysis

In the process of UVMR analysis, the Wald ratio test was meticulously employed to scrutinize individual IVs. Subsequently, causal relationships involving multiple IVs (≥2) were systematically explored using the multiplicative random-effects inverse-variance-weighted (IVW) method. A fixed-effects model was implemented when the heterogeneity index I^2^ was less than 50%, further enhanced by the integration of both MR-Egger and weighted median approaches. In this framework, the IVW weighting has a direct correlation with the Wald ratio estimate for each SNP and an inverse relationship with the variance estimate of the Wald ratio for the specific SNP (31). In scenarios where all genetic variations are classified as valid, IVW yields both dependable and efficient estimations. Conversely, the weighted median method proves superior when a minimum of half of the genetic variations are evaluated as invalid, and the MR-Egger method is called upon when all genetic variations are considered invalid (32). Finally weighted mode and simple mode are used as complementary methods to increase the confidence of the results.

To ensure reliable MR estimates, a series of sensitivity analyses were conducted. Cochran’s Q test was utilized to evaluate the heterogeneity of each genetic variant, with a *P*-value under 0.05 indicating significant heterogeneity among the selected SNPs (33). Directional pleiotropy in our MR study was scrutinized using MR-Egger regression (34), and a *P*-value below the 0.05 threshold concerning the MR-Egger’s intercept may suggest significant directional pleiotropy. Although the MR-Egger method has relatively lower accuracy, its intercept can indicate the presence of directional pleiotropy (35). The MR-PRESSO method was employed to identify potential outliers and investigate horizontal pleiotropy, which is presumed if the global P-value falls below 0.05 (36). Any outliers would be removed to enhance the accuracy of the correction. Lastly, a leave-one-out analysis was conducted to evaluate the impact of individual SNPs on the overall results (37).

The study calculated R^2^ = 2×MAF×(1-MAF)×beta^2^, where MAF indicates minor allele frequency, of each instrumented SNP and summed the values for the coefficient necessary for the power calculator (38). We calculated the statistical power using the mRnd website (https://shiny.cnsgenomics.com/mRnd/) (39).

## Results

### Genetic instrument selection and genetic correlation between phenotypes

The research report indicates that the F-statistics for all IVs exceeded 100, signifying a substantial reduction in bias due to weak instruments. In both forward and reverse MR analyses, 89 (**Supplementary File 2-Table 1**) and 7 (**Supplementary File 2-Table 2**) SNPs were selected as IVs, accounting for 7.92% and 27.93% of the explained variance, respectively. We possess 75% statistical power to detect a correlation between SUA and DN, with an OR of 1.17.

LDSC analysis revealed a significant genetic correlation between SUA and DN (r_g_ = 0.293, *P* = 2.60×10^-5^), and the SNP-based heritability (h²) for SUA and DN were found to be 9.64% and 1.05%, respectively.

### Association of genetically predicted SUA with DN

**Figure 2:**
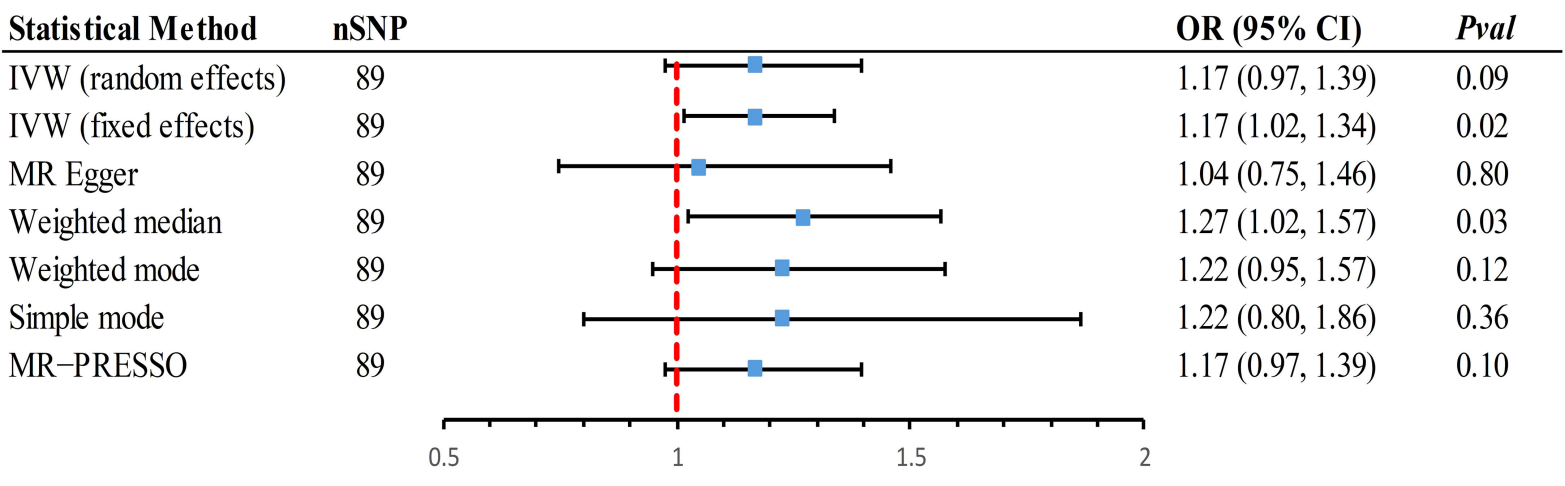
Mendelian randomization association of genetically predicted SUA with DN using different statistical models. Odds ratios are scaled per 1 mg/dL increase in the genetically predicted serum urate level. IVW, inverse-variance-weighted method; MR, Mendelian randomization; MR-PRESSO, MR-pleiotropy residual sum and outlier; OR, odds ratio; CI, confidence interval; SUA, Serum urate acid; DN, Diabetic Neuropathy.

In the forward MR analysis, scatter plots vividly displayed a positive correlation between SUA and DN (**Supplementary File 1-Figure 1**). Since an I^2^ of 42% (less than 50%) was detected, a fixed-effects model was also chosen to estimate the causal effect. The main method, IVW (fixed effects model), indicated that each increase of 1 mg/dL in SUA would increase the risk of DN by 17% (OR = 1.17, 95% CI 1.02~1.34, *P* = 0.02) (**Figure 2**). A random-effects result did not detect a causal relationship (OR = 1.17, 95% CI 0.971.39, *P* = 0.09). Further, the weighted median estimate was consistent with the primary method (OR = 1.27, 95% CI 1.02~1.57, *P* = 0.03), considering the main results robust, albeit with a wide 95%CI.

Sensitivity analyses confirmed the robustness of these results (**Supplementary File 2-Table 3**). MR-PRESSO did not find any outliers, but there was unavoidable horizontal pleiotropy (*P* < 0.05). Leave-one-out analysis further substantiated that the causal relationship was not influenced by any individual SNP. The symmetry of the funnel plot was maintained, and after performing the steiger test, the causal relationship remained significant, bolstering the stability of our findings (**Supplementary File 1-Figure 1**).

In the reverse MR analysis (**Supplementary File 1-Figure 2**), the primary analytical method indicated that there was no causal association between genetically predicted DN and SUA (OR = 1.00, 95% CI 0.98~1.01, *P* = 0.97) (**Supplementary File 2-Table 4**). The estimates from **Supplemental Methods** were consistent with this finding. A series of sensitivity analyses revealed no outliers, pleiotropy, or heterogeneity, and the results were not distorted by any individual SNP. Thus, the findings are robust (**Supplementary File 2-Table 3**).

### MVMR analysis adjusted for ten confounders

**Figure 3:**
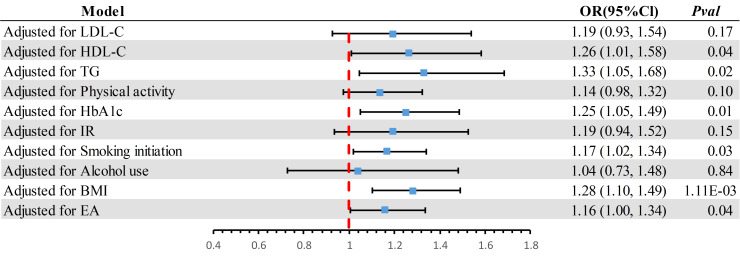
The effect of potential confounders was adjusted individually. BMI, body mass index; CI, confidence interval; LDL-C, Low Density Lipoprotein Cholesterol; HDL-C, High Density Lipoprotein Cholesterol; TG, Triglyceride; IR, insulin resistance; EA, Education Attainment; HbA1c, Glycated Hemoglobin A1c.

In the MVMR analysis (**Figure 3**), after adjusting for potential confounders such as LDL-C, physical activity, IR, and alcohol use, the causal association between SUA and DN was no longer significant (*P* > 0.05). This suggests that the partial causal relationship of SUA on DN might be mediated through these phenotypes.

## Discussion

This study conducted a comprehensive MR analysis to delve into the relationship between genetic predisposition to SUA levels and DN. The MR findings corroborate prior epidemiological studies (11–14), establishing a causal link between elevated SUA levels and an increased risk of DN. Additionally, no reverse causal association was identified between DN and SUA. Further MVMR analysis indicated that physical activity, LDL-C, IR, and alcohol use may mediate a portion of this causal relationship.

Previous research has suggested a connection between SUA and DN, with findings indicating an association between SUA and a heightened risk of DN (40–43). A meta-analysis involving 12 studies with 6,134 patients supported that hyperuricemia independently correlates with an elevated risk of peripheral neuropathy amongst patients diagnosed with type 2 diabetes (44). However, prior studies have reported conflicting findings regarding the connection between generalized SUA and DN (15, 16). Specifically, a cross-sectional study found no association between hyperuricemia and diabetic polyneuropathy (45). Moreover, SUA is a recognized risk factor for diverse conditions such as Alzheimer’s disease, sudden cardiac death, and gout.

SUA acts as a surrogate marker for oxidative stress, where environments with elevated uric acid can trigger oxidative stress, intimately tied with inflammation (46, 47). Oxidative stress epitomizes a disparity between reactive oxygen species (ROS) and the body’s inherent antioxidant defense mechanism (48). Chronic hyperglycemic disorders can incite protein glycation and stimulate an increase in free radicals, including ROS. At the same time, the antioxidant defense system suffers impairment in these hyperglycemic conditions (49, 50). Additionally, SUA promotes inflammatory responses and activates inflammatory pathways (51). A specific study revealed that uric acid innately incites inflammation by direct activation of the NLRP3 inflammasome, resulting in an upsurge in IL-1β and IL-18 expression within lung macrophages (52). Uric acid can penetrate the blood-brain barrier and initiate an inflammatory response through the activation of the NFκB-mediated inflammation pathway (53). High uric acid levels might aggravate these conditions, leading to nerve cell damage and inflammatory responses that could precipitate DN. Meanwhile, Uric acid is observed to interfere with nitric oxide (NO) synthesis, inciting vascular endothelial dysfunction (54). NO is known to dilate blood vessels and enhance local tissue perfusion (55). NO deficiency may contribute to nerve ischemia and hypoxia, further intensifying the progression of diabetic neuropathy. Recent findings have demonstrated uric acid’s role in influencing insulin secretion by escalating oxidative stress in β cells (56), thereby triggering insulin resistance. This insulin resistance is closely associated with the development of neuropathy and could deteriorate glycemic control further, exacerbating oxidative stress and inflammatory responses.

Considering the intrinsic limitations of observational studies, these investigations might not eliminate the influence of unobserved confounding factors and reverse causality. Observational studies typically emphasize correlation over causation. Despite some data suggesting a link, the causal relationship between SUA and DN is yet to be definitively established. We employed MR analysis to explore the genetic basis of the causal relationship between SUA and DN, aiming to negate these biases and confounders. The results of this analysis support that SUA is a risk factor for DN. As blood uric acid levels can be modified, uric acid-lowering therapy may offer preventive or therapeutic advantages in patients with DN.

Further MVMR analyses underscored the significance of physical activity, LDL-C, IR, and alcohol use. Firstly, physical activity impacts metabolic health and systemic inflammatory responses. Regular exercise has been shown to enhance insulin sensitivity, a notion that’s also supported by a NHANES study conducted by Yajuan Lin et al. (57). Secondly, Hui Zhang and colleagues have demonstrated that elevated LDL-C can instigate endothelial dysfunction and inflammation, exacerbating the pathophysiology of diabetic neuropathy (58). Additionally, a meta-analysis by Dovell et al. has pointed out a link between increased SUA and heightened IR (59), which further amplifies the risk of DN. Lastly, chronic alcohol consumption not only elevates serum urate levels but can also directly impact glucose metabolism and intensify the risk of neuropathic complications (60, 61). Hence, this suggests a multifaceted approach to the management of diabetic neuropathy, considering these factors collectively.

Our study possesses several strengths. This MR study is the first to establish a causal relationship between SUA and DN. All the SNPs set as IVs were derived from the European population, thereby reducing the likelihood of population stratification bias and enhancing the validity of the bidirectional MR assumption. Our robust tools employed in this study (e.g., an F statistic significantly exceeding 10) should mitigate potential bias from sample overlap. However, our study is not without its constraints. Some of the first selected exposures originated from the UKB cohort and the lack of other GWAS studies prevented a positive control analysis. Finally, due to the availability of only summary-level GWAS data, further subgroup analyses were precluded.

## Conclusion

To encapsulate, our MR study establishes a causal link between elevated SUA levels and increased risk of DN, with no evidence for a reverse association. The potential therapeutic role of urate-lowering strategies in DN management warrants further investigation.

## Data availability statement

The original contributions presented in the study are included in the article/**Supplementary Material**. Further inquiries can be directed to the corresponding author.

## Ethics statement

Ethical approval was not required for the study involving humans in accordance with the local legislation and institutional requirements. Written informed consent to participate in this study was not required from the participants or the participants’ legal guardians/next of kin in accordance with the national legislation and the institutional requirements.

## Author contributions

YZ: Writing – review & editing, Writing – original draft. ZT: Writing – review & editing. LT: Conceptualization, Writing – original draft. YW: Writing – original draft, Validation. LL: Writing – review & editing.
