# Supplementary material for: Serum uric acid and risk of diabetic neuropathy: a genetic correlation and mendelian randomization study
Source: Front Endocrinol (Lausanne). 2023 Nov 15;14:1277984. doi: 10.3389/fendo.2023.1277984 (PMC10684953; doi:10.3389/fendo.2023.1277984)
Supplement: Supplementary file 1 [file DataSheet_1.docx]

Contents

1.[Description of summary statistics data sources 1](#_Toc147060007)

[1.1 GWAS of diabetic neuropathy (DN) by FinnGen consortium. 1](#_Toc147060008)

1.2 GWAS of serum urate acid (SUA) by CKDGen consortium. 1

[1.3 GWAS of Triglyceride, Low Density Lipoprotein Cholesterol and High Density Lipoprotein Cholesterol by Cristen J Willer et al 2](#_Toc147060010)

[1.4 GWAS of Glycated Hemoglobin A1c (HbA1c) by MAGIC 2](#_Toc147060011)

[1.5 GWAS of insulin resistance (IR) by Dupuis J et al. 3](#_Toc147060012)

[1.6 GWAS of Smoking initiation by GSCAN 3](#_Toc147060013)

[1.7 GWAS of alcohol use by PGC consortium 4](#_Toc147060014)

[1.8 GWAS of Body Mass Index (BMI) by GIANT consortium 4](#_Toc147060015)

[1.9 GWAS of Educational Attainment (EA) by Lee et al. 5](#_Toc147060016)

[1.10 GWAS of physical activity by Howe LJ et al. 5](#_Toc147060016)

2. [Supplementary-Fig. 1 Forest plot (A), leave-one-out analysis (B), scatter plot (C), and funnel plot (D) of the effect of SUA on DN 7](#_Toc147060017)

3. [Supplementary-Fig. 2 Forest plot (A), leave-one-out analysis (B), scatter plot (C), and funnel plot (D) of the effect of DN on SUA 8](#_Toc147060018)

[Reference 9](#_Toc147060022)

**1.Description of summary statistics data sources**

**1.1** **GWAS of diabetic neuropathy (DN) by FinnGen consortium(https://www.finngen.fi/en)**

Diabetic neuropathy data pertaining to diabetes complications were obtained from the latest R9 version of the FinnGen consortium. The consortium amalgamates summary-level data, representing a broad spectrum of diabetes-related complications within an expansive population cohort. The FinnGen consortium is distinctive for its meticulous integration of genomic data with rich health insights derived from national health registries, ensuring a gold standard in disease classification.

DN(2,843 cases / 271,817 controls) is defined by the International Classification of Diseases (ICD-10). Within the ICD-10, its parent code is categorized as E1[0-4]4. The disease is described as follows: A chronic, pathological complication associated with diabetes mellitus, where nerve damages are incurred due to diabetic microvascular injury involving small blood vessels that supply these nerves, resulting in peripheral and/or autonomic nerve dysfunction.( https://risteys.finregistry.fi/endpoints/DM_NEUROPATHY)

**1.2 GWAS of serum urate acid (SUA) by CKDGen consortium**(1)**.**

Serum urate acid is a biomarker that has been extensively studied in the context of gout and other metabolic disorders. In this GWAS study, a trans-ethnic meta-analysis of GWAS of serum urate among 457,690 individuals was performed, and 183 associated loci were identified that improve gout risk prediction in an independent sample of 334,880 UK Biobank (UKBB) participants .

The study found that serum urate showed significant genetic correlations with many cardio-metabolic traits, with genetic causality analyses supporting a substantial role for pleiotropy . The genetic urate risk score led to significant improvements of gout risk prediction among UKBB participants, with 3.5% having a risk of gout comparable to a Mendelian disease effect size . The largest GWAS meta-analyses performed to date identified 28 loci among European ancestry (EA) and 27 among Japanese individuals. Many genes in the associated loci encode renal and intestinal urate transporters or their regulators, while others are relevant to glucose and lipid metabolism, functions of the liver, where uric acid is generated .

The study also evaluated the genetic correlation of serum urate with hundreds of cardio-metabolic traits and diseases, and used a recently developed latent causal variable model to examine the contribution of causality . Tissue and cell type-specific enrichment analyses supported kidney and liver, the sites of urate excretion and generation, as key target tissues. Comprehensive fine-mapping and co-localization analyses with gene expression across 47 tissues delivered an extensive list of target genes and SNPs for follow-up studies, of which HNF4A p.Thr139Ile was experimentally confirmed as a functional allele involved in transcriptional regulation of urate homeostasis .

Overall, this GWAS study provides a comprehensive analysis of serum urate acid and its genetic correlations with various cardio-metabolic traits and diseases. The identification of associated loci and target genes provides valuable insights into the underlying mechanisms of urate homeostasis and metabolic disorders, and may lead to the development of new therapeutic strategies for gout and related diseases.

**1.3 GWAS of Triglyceride, Low Density Lipoprotein Cholesterol and High Density Lipoprotein Cholesterol by** **Cristen J Willer *et al***(2)***.***

The GWAS study analyzed in this article aimed to identify new loci and refine known loci associated with lipid levels, including low-density lipoprotein (LDL) cholesterol, high-density lipoprotein (HDL) cholesterol, triglycerides, and total cholesterol. The study examined 188,578 individuals using genome-wide and custom genotyping arrays and identified and annotated 157 loci associated with lipid levels at P < 5×10^-8^, including 62 loci not previously associated with lipid levels in humans. Of the 62 novel loci, 24 demonstrated the strongest evidence of association with HDL cholesterol, 15 with LDL cholesterol, 8 with triglyceride levels, and 15 with total cholesterol. The effects of newly identified loci were generally smaller than in earlier GWAS. For the 62 newly identified variants, trait variance explained in the Framingham offspring were 1.6% for HDL cholesterol, 2.1% for triglycerides, 2.4% for LDL cholesterol, and 2.6% for total cholesterol .

To investigate connections between the new loci and known lipid biology, the study catalogued genes within 100 kb of the peak associated SNPs and searched PubMed and OMIM for occurrences of these gene names and their aliases in the context of relevant keywords. After manual curation, the study identified at least one strong candidate in 32 of the 62 loci (52%). For the remaining 30 loci, the study found no literature support for the role of a nearby gene on blood lipid levels . Among independent variants (r^2^ < 0.1) with P < 0.1 in the GWAS-only analysis, a significant excess were concordant in direction of effect for HDL (62.9% in 1,847 SNPs, P < 10^-16^), LDL (58.6% of 1,730 SNPs, P < 10^-16^), triglyceride levels (59.1% of 1,783 SNPs, P < 10^-16^), and total cholesterol (61.0% of 1,904 SNPs, P < 10^-16^), suggesting many additional loci to be discovered in future studies .

**1.4 GWAS of Glycated Hemoglobin A1c (HbA1c) by** **MAGIC**(3)***.***

Glycated Hemoglobin A1c (HbA1c) is a biomarker that reflects the average blood glucose levels over the past 2-3 months. It is widely used as a diagnostic tool for diabetes and as a monitoring tool for glycemic control in diabetic patients. In this summary statistics data source, we present the results of a genome-wide association study (GWAS) that aimed to identify common genetic variants associated with HbA1c levels in nondiabetic individuals of European ancestry.

The GWAS included up to 46,368 participants from 23 studies and 8 cohorts, and used de novo genotyped single nucleotide polymorphisms (SNPs) to test for associations with HbA1c levels. The study employed inverse-variance meta-analysis to combine the results of individual studies and tested mediation by glycemia using conditional analyses. The global effect of HbA1c loci was estimated using a multilocus risk score, and net reclassification was used to estimate genetic effects on diabetes screening. The results of the GWAS identified 10 loci that reached genome-wide significant association with HbA1c levels, including six new loci near FN3K, HFE, TMPRSS6, GCK, ANK1, and SPTA1. The lead SNP for FN3K was rs1046896 (P=1.6×10-^26^), for HFE was rs1800562 (P=2.6×10^-20^), for TMPRSS6 was rs855791 (P=2.2×10^-13^), for GCK was rs730497 (P=1.1×10^-10^), for ANK1 was rs4737009 (P=1.2×10^-9^), and for SPTA1 was rs2779116 (P=1.3×10^-9^). The other four loci were previously identified in other GWAS studies.

The study also found that the net reclassification of the population-level effect size of the seven nonglycemic HbA1c-associated SNPs was equivalent to reclassification of about 2% of a European ancestry population sample according to HbA1c-determined diabetes status. This suggests that the identified genetic variants have a modest effect on diabetes classification, but may still be useful for improving diabetes screening and diagnosis.

**1.5 GWAS of insulin resistance (IR) by Dupuis J et al**(4)**.**

The GWAS of insulin resistance and related traits analyzed in this study included data from 21 cohorts, comprising up to 46,186 non-diabetic participants of European descent informative for fasting glucose (FG), and 20 GWAS including up to 38,238 non-diabetic individuals informative for fasting insulin (FI), as well as the surrogate estimates of β-cell function (HOMA-B) and insulin resistance (HOMA-IR) derived from fasting variables by homeostasis model assessment.

The study analyzed ~2.5 million directly genotyped or imputed autosomal SNPs from these cohorts, and identified 16 loci associated with fasting glucose and insulin resistance. Nine of these loci were novel, and five of the novel loci also demonstrated genome-wide significant evidence for association between the glucose-raising allele and T2D risk in up to 40,655 cases and 87,022 non-diabetic controls. The study found that HOMA-B and HOMA-IR have comparable heritability estimates, and their correlation is significant. However, the genetic architecture of each trait may be distinct, with more modest effects, fewer loci, rarer variants, or a stronger environmental modification underlying HOMA-IR. In addition, HOMA-IR is an imperfect estimate of global insulin resistance, as it addresses mostly hepatic sensitivity to insulin and is partially affected by β-cell function.

The study also identified likely biological candidate genes that influence signal transduction, cell proliferation, development, glucose-sensing, and circadian regulation within the identified loci. These genes provide new insights into the underlying biology of insulin resistance and related traits, and may reveal novel pathways for diabetes therapeutics.

**1.6 GWAS of Smoking initiation by** **GSCAN**(5)***.***

The study aimed to identify genetic variants associated with smoking behavior and related phenotypes. The data sources used in the study were collected from multiple studies and included both related and unrelated individuals. The summary statistics data sources for smoking included GWAS meta-analyses of five substance use phenotypes. These phenotypes were smoking initiation, smoking cessation, age of smoking initiation, cigarettes per day, and drinks per week. The data sources were obtained from studies that genotyped participants on genome-wide arrays and imputed their genotypes to the Haplotype Reference Consortium using either Minimac3 or IMPUTE2.

The studies used RVTESTS to generate GWAS summary statistics for each sample. For studies composed primarily of related individuals, covariates including genetic principal components were regressed out under a linear model. The residuals were then inverse-normalized (except for 23andMe) and tested for an additive effect of each variant under a linear mixed model with a genetic kinship matrix. Family studies followed this analysis for all phenotypes, even binary phenotypes such as smoking initiation and cessation. For studies of entirely unrelated individuals, the same analysis was followed for quasi-continuous phenotypes (AgeSmk, CigDay, DrnkWk). However, for binary phenotypes (SmkInit and SmkCes), additive genetic effects were estimated under a logistic model. The GWAS summary statistics data sources for smoking can be downloaded from the world wide web. Association results for all SNPs that passed quality-control filters in a GWAS meta-analysis of each of the five substance use phenotypes are provided. However, the research participants from 23andMe are excluded from the data.

**1.7 GWAS of alcohol use by** **PGC consortium**(6)***.***

The study used quantitative measures from the AUDIT from two population-based cohorts of European ancestry, the UK Biobank and 23andMe, and performed a GWAS meta-analysis. The study also explored the shared genetic basis between these measures and other substance use, psychiatric, and behavioral traits.

The GWAS meta-analysis of the AUDIT total score identified 10 associated risk loci. These loci include ADH1B, ADH1C, ADH5, KLB, and GCKR, which have previously been associated with both pharmacokinetic and pharmacodynamic factors that influence alcohol consumption. The genetic overlap between alcohol consumption and diagnosed DSM-IV alcohol dependence is moderate, which reinforces the notion that alcohol consumption cannot be used as a surrogate for alcohol use disorders. Two additional GWAS analyses were performed, a GWAS for AUDIT scores on items 1–3, which focus on consumption (AUDIT-C), and for scores on items 4–10, which focus on the problematic consequences of drinking (AUDIT-P). The GWAS meta-analysis of AUDIT-C identified 8 associated risk loci, while the GWAS meta-analysis of AUDIT-P identified 12 associated risk loci.

**1.8 GWAS of** **Body Mass Index (BMI) by** **GIANT consortium**(7)***.***

The summary statistics data sources for the genome-wide association study (GWAS) of Body Mass Index (BMI) were obtained from two previous studies: Wood et al. and Locke et al. Before conducting the meta-analysis with the UK Biobank (UKB) data, the researchers filtered out SNPs that did not match the pairs of alleles in the HRS and UKB and those that had reported allele frequencies that were too different from that calculated using unrelated participants of HRS. After filtering the data, the researchers performed a fixed-effect inverse variance weighted meta-analysis using the software METAL . This approach allowed them to combine the summary statistics from the two previous studies with the GWAS of height and BMI performed in ∼450 000 UK Biobank participants of European ancestry.

The combined GWAS meta-analysis reached N ∼700 000 individuals and substantially increased the number of GWAS signals associated with BMI. The researchers identified 941 near-independent SNPs associated with BMI at a revised genome-wide significance threshold of P < 1 × 10^-8^, including 751 BMI-associated SNPs located within loci not previously identified by these two GWAS. The near-independent genome-wide significant SNPs explained ∼6.0% of the variance of BMI in an independent sample from the Health and Retirement Study (HRS). This finding suggests that there are likely many more genetic factors that influence BMI that have yet to be identified.

To control for potential confounding variables, the researchers performed LDSC to quantify the level of confounding in GWAS due to population stratification as well as quantifying the genetic correlation between BMI and other traits. This approach allowed them to identify potential confounding variables and control for them in their analysis.

**1.9 GWAS of Educational Attainment (EA) by Lee *et al***(8)***.***

In the comprehensive analysis, data was converted from 71 distinct European ancestry cohorts. Each cohort's subjects were examined based on their highest educational qualifications, which were subsequently aligned with the International Standard Classification of Education (ISCED) categories. The educational attainment phenotype was meticulously constructed by calculating the corresponding years of education for each ISCED classification. Across all cohorts, the sample-size-weighted mean and standard deviation for the educational attainment phenotype were found to be 16.8 and 4.2 years, respectively.

The analytical approach was devised to execute association evaluations at the cohort level, incorporating adjustments for variables such as age, sex, their interaction, and specific genetic principal components. The meta-analysis, weighted by sample size, yielded association estimates for nearly 10 million autosomal Single Nucleotide Polymorphisms (SNPs) that satisfied the quality criteria across all cohorts. Of these, 1,271 SNPs were identified as approximately independent (r^2^ < 0.1) and achieved genome-wide significance, with a p-value less than (P < 5 × 10^-8^).

A rigorous quality-control procedure was uniformly applied to the results of each cohort. Genotypic imputations were performed utilizing either the 1,000 Genomes Project Phase 3 European reference panel or an expanded reference panel furnished by the Haplotype Reference Consortium. Filters at the subject level were imposed to exclude non-European individuals, those with inadequate genotyping rates, and genetic anomalies. Furthermore, subjects with educational attainment phenotypes ascertained before age 30 were excluded from the study. The genotype filtering process was comprehensive, encompassing the elimination of INDELs, non-autosomal variants, SNPs with recognized strand complications in imputation methodologies, SNPs exhibiting suboptimal imputation precision, and those with minor allele counts less than 25. Exclusions also extended to SNPs with erroneous or duplicated chromosomal coordinates, missing values for particular variables, or alleles inconsistent with the reference panel.

**1.10 GWAS of physical activity by** **Howe LJ *et al***(9)***.***

The authors of this study have used summary statistics data from various sources to investigate the impact of these effects on genetic associations. The European meta-analysis summary statistics for both the within-sibship and population GWAS models are publicly available on OpenGWAS. The relevant GWAS IDs in OpenGWAS are ieu-b-4813 to ieu-b-4860. For example, within-sibship GWAS estimates for height are in https://gwas.mrcieu.ac.uk/datasets/ieu-b-4813/. These summary statistics data provide a wealth of information on the genetic associations of various phenotypes.

The authors have also used data from 178,076 individuals (with one or more genotyped siblings) from 77,832 sibships in 19 studies. Sample sizes for individual phenotypes ranged from 13,375 to 163,748 (median: 82,760, mean: 79,794). More information on sample sizes from individual cohorts and for each phenotype is contained in Supplementary Table 1. The within-sibship models used deviations of the individual’s genotype from the mean genotype within the sibship. The within-sibship model includes the mean sibship genotype as a covariate to capture the between-family contribution of the SNP. The authors have also used data from the China Kadoorie Biobank to investigate the impact of within-sibship shrinkage estimates.


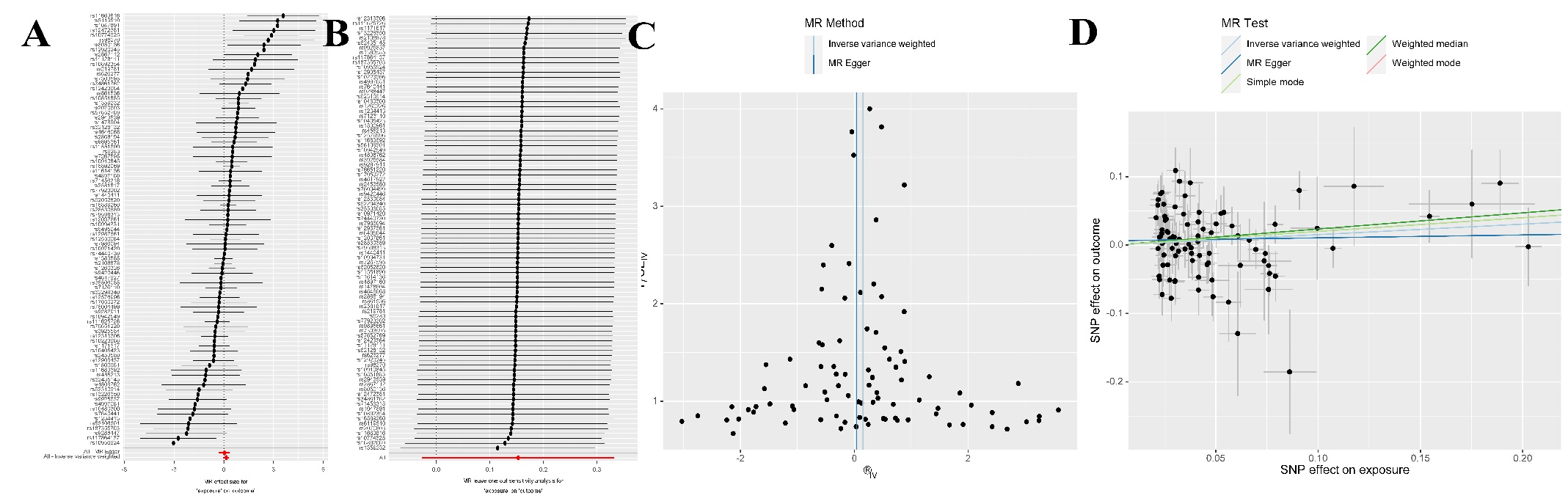


**2.** **Supplementary-Fig. 1** Forest plot (A), leave-one-out analysis (B), scatter plot (C), and funnel plot (D) of the effect of SUA on DN


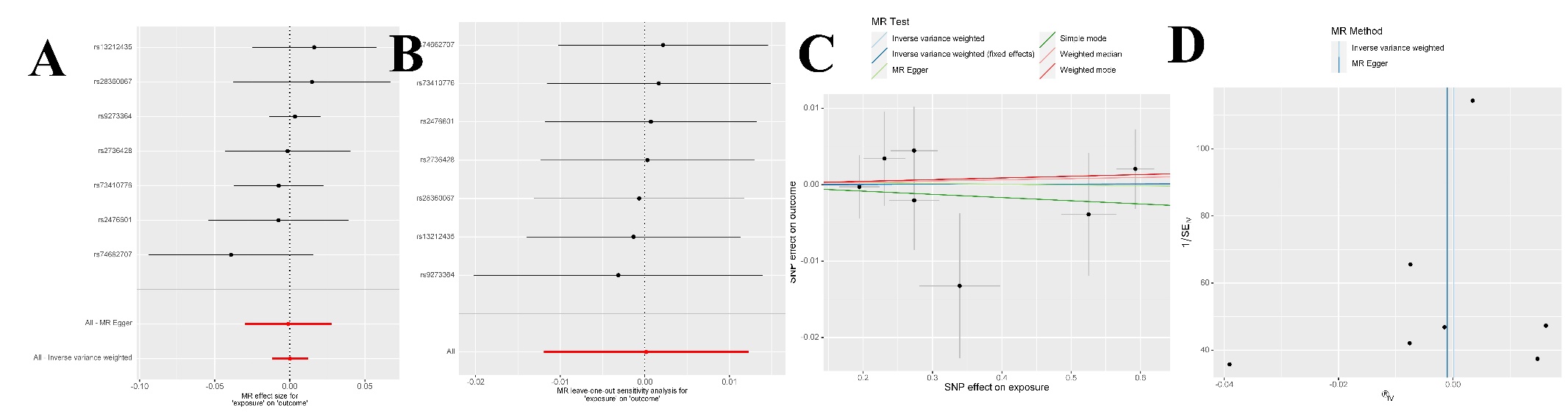


**3. Supplementary-Fig. 2** Forest plot (A), leave-one-out analysis (B), scatter plot (C), and funnel plot (D) of the effect of SUA on DN

**Reference**

1. Tin A, Marten J, Halperin Kuhns VL, Li Y, Wuttke M, Kirsten H, Sieber KB, Qiu C, Gorski M, Yu Z, et al. Target genes, variants, tissues and transcriptional pathways influencing human serum urate levels. *Nat Genet* (2019) 51:1459–1474. doi: 10.1038/s41588-019-0504-x

2. Willer CJ, Schmidt EM, Sengupta S, Peloso GM, Gustafsson S, Kanoni S, Ganna A, Chen J, Buchkovich ML, Mora S, et al. Discovery and refinement of loci associated with lipid levels. *Nat Genet* (2013) 45:1274–1283. doi: 10.1038/ng.2797

3. Soranzo N, Sanna S, Wheeler E, Gieger C, Radke D, Dupuis J, Bouatia-Naji N, Langenberg C, Prokopenko I, Stolerman E, et al. Common variants at 10 genomic loci influence hemoglobin A₁(C) levels via glycemic and nonglycemic pathways. *Diabetes* (2010) 59:3229–3239. doi: 10.2337/db10-0502

4. Dupuis J, Langenberg C, Prokopenko I, Saxena R, Soranzo N, Jackson AU, Wheeler E, Glazer NL, Bouatia-Naji N, Gloyn AL, et al. New genetic loci implicated in fasting glucose homeostasis and their impact on type 2 diabetes risk. *Nat Genet* (2010) 42:105–116. doi: 10.1038/ng.520

5. Liu M, Jiang Y, Wedow R, Li Y, Brazel DM, Chen F, Datta G, Davila-Velderrain J, McGuire D, Tian C, et al. Association studies of up to 1.2 million individuals yield new insights into the genetic etiology of tobacco and alcohol use. *Nat Genet* (2019) 51:237–244. doi: 10.1038/s41588-018-0307-5

6. Sanchez-Roige S, Palmer AA, Fontanillas P, Elson SL, Adams MJ, Howard DM, Edenberg HJ, Davies G, Crist RC, Deary IJ, et al. Genome-wide association study meta-analysis of the Alcohol Use Disorder Identification Test (AUDIT) in two population-based cohorts. *Am J Psychiatry* (2019) 176:107–118. doi: 10.1176/appi.ajp.2018.18040369

7. Yengo L, Sidorenko J, Kemper KE, Zheng Z, Wood AR, Weedon MN, Frayling TM, Hirschhorn J, Yang J, Visscher PM, et al. Meta-analysis of genome-wide association studies for height and body mass index in ∼700000 individuals of European ancestry. *Hum Mol Genet* (2018) 27:3641–3649. doi: 10.1093/hmg/ddy271

8. Lee JJ, Wedow R, Okbay A, Kong E, Maghzian O, Zacher M, Nguyen-Viet TA, Bowers P, Sidorenko J, Karlsson Linnér R, et al. Gene discovery and polygenic prediction from a genome-wide association study of educational attainment in 1.1 million individuals. *Nat Genet* (2018) 50:1112–1121. doi: 10.1038/s41588-018-0147-3

9. Howe LJ, Nivard MG, Morris TT, Hansen AF, Rasheed H, Cho Y, Chittoor G, Ahlskog R, Lind PA, Palviainen T, et al. Within-sibship genome-wide association analyses decrease bias in estimates of direct genetic effects. *Nat Genet* (2022) 54:581–592. doi: 10.1038/s41588-022-01062-7
